# Redox and actin, a fascinating story

**DOI:** 10.1016/j.redox.2025.103630

**Published:** 2025-04-12

**Authors:** Pascal J. Goldschmidt-Clermont, Brock A. Sevilla

**Affiliations:** a University of Miami, Miller School of Medicine, Miami, FL, USA; b United States Army Career Skills Program, USA

**Keywords:** Actin, Profilin, ADF/Cofilin, MICAL, Ras, Rac1, EGF-Receptor, PDGF-Receptor, Superoxide, Hydrogen peroxide, Reactive oxygen species, NADPH-Oxidase, NOX-1, NOX-2, Phosphatases, Cell motility, Cytoskeleton, Lamellipodium, Phosphatidylinositol 4,5 bisphosphate, Phospholipase C-y1, Membranes, Bubbles, Kaposi's sarcoma, Cancer, Atherosclerosis, Tissue repair, Regeneration, Inflammation

## Abstract

Actin is an extraordinarily complex protein whose functions are essential to cell motility, division, contraction, signaling, transport, tissular structures, DNA repair, and many more cellular activities critical to life for both animals and plants. It is one of the most abundant and conserved proteins and it exists in either a soluble, globular (monomeric, G-actin) or an insoluble, self-assembled (polymerized or filamentous actin, F-actin) conformation as a key component of the cytoskeleton. In the early 1990's little, if anything, was known about the impact of reactive oxygen species (ROS) on the biology of actin except that ROS could disrupt the actin cytoskeleton. Instructively, G-actin is susceptible to alteration by ROS, and thus, purification of G-actin is typically performed in the presence of strong antioxidants (like dithiothreitol) to limit its oxidative degradation. In contrast, F-actin is a more stable conformation and thus actin can be kept relatively intact in purified preparations as filaments at low temperature for extended periods of time. Both G- and F-actin interact with a myriad of intracellular proteins and at least with a couple of extracellular proteins, and these interactions are essential to the many actin functions. This review will show how, over the past 30 years, our understanding of the role of ROS for actin biology has evolved from noxious denaturizing agents to remarkable regulators of the actin cytoskeleton in cells and consequent cellular functions.

## 1 Introduction

Actin, one of the most abundant and essential proteins in eukaryotic and plant cells, plays a critical role in cell structure, movement, division, and signaling [1,2]. In the cytoplasm of non-muscle cells, actin forms a solid phase (microfilaments), a major component of the cytoskeleton [1]. The dynamic polymerization (near the advancing edge) and depolymerization (close to the nucleus) of actin filaments in response to extracellular and intracellular signals, together called treadmilling, are central to various cellular functions like cell motility [2,3]. Because in non-muscle cells, the cytoplasmic concentration of monomeric actin is well above the critical concentration of actin at both ends of filament, it is difficult to understand how actin filaments can be treadmilling in motile cells.

Redox chemistry, the study of oxidation-reduction reactions, refers to the processes by which electrons are transferred between atoms within molecules, often influencing the oxidation states of proteins [4,5]. Within the context of cellular function, reactive oxygen species (ROS) and reactive nitrogen species (RNS) are critical players in redox signaling and cellular regulation [6,7]. Low levels of ROS and RNS can act as important signaling molecules, whereas excessive production can lead to damage of cellular components, including proteins, lipids, and DNA [[5], [6], [7], [8]].

**Fig. 1:**
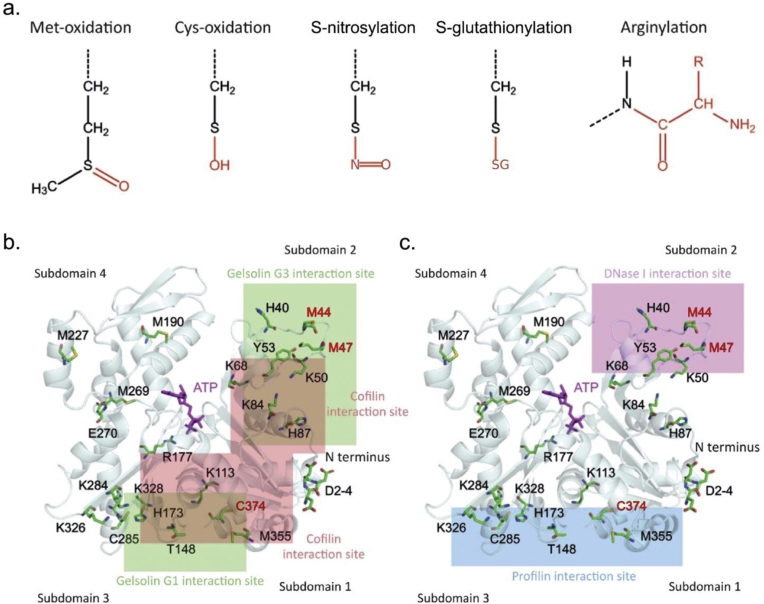
Adapted from Varland et al. (Fig. 1) [10]. A. Structures of selected actin post translation modifications (PTMs): methylation, acetylation, arginylation, phosphorylation, methionine (met) oxidation, cysteine (cys) oxidation, S-nitrosylation, and S-glutathionylation (SG represents glutathione). B. Interaction sites for specific actin binding proteins (ABPs) are represented by the green (Gelsolin G1 & G3 interaction sites), burgundy (Cofilin), pink (DNase I), and blue (Profilin) boxes. Key actin residues that are modified by PTMs are indicated by red text (M44 and M47 by Met-oxidation; C374 by Cys-oxidation, including nitrosylation and glutathionylation); this demonstrates how residues in the aforementioned ABP binding sites are the subject of PTMs that have significant effects on actin's ability to interact with ABPs.

Thirty years ago, very little was known about the posttranslational modification (PTM) of actin and its binding proteins, except for tyrosine, serine, and threonine phosphorylation, methylation, and ubiquitination. Actin biology was believed to be regulated essentially by some actin-binding proteins, nucleotides, and salts like calcium and magnesium [9]. And while it is true that binding proteins, nucleotides, and salts direct much of the biology of actin, the regulation of actin by redox chemistry and other PTM is particularly significant for actin response to cell signaling, oncogenes, and other environmental changes, where ROS and RNS can regulate actin dynamics {Fig. 1}. For instance, certain signaling pathways involve the modification of actin by oxidative modifications, such as oxidized actin, which can influence the stability and polymerization of actin filaments [5,10]. Similar PTM of actin-binding proteins can further refine cellular responses. This interplay is crucial in both physiological processes (such as cell movement during wound healing) and pathophysiological conditions (such as cancer growth, cardiovascular disease, and neurodegenerative disorders) [5,[10], [11], [12], [13], [14]]. Thus, understanding the relationship between actin dynamics and redox chemistry offers crucial insights into the regulation of normal cellular activities and how their dysregulation can lead to disease.

## 2 G-actin partners in cells maintain actin's ability to polymerize in the presence of ROS

In cells, where oxidants and other post-translational modification factors are ubiquitous, it is remarkable that G-actin can exist without denaturation and maintains intact functionality [1,2,15,16]. Of course, cells also contain powerful antioxidants that can buffer the oxidation of actin; however, upon exposure to stimuli like epidermal growth factor (EGF) or platelet-derived growth factor (PDGF), specific regions within the cell can transition to a locally oxidant environment [[17], [18], [19], [20], [21]].

**Fig. 2:**
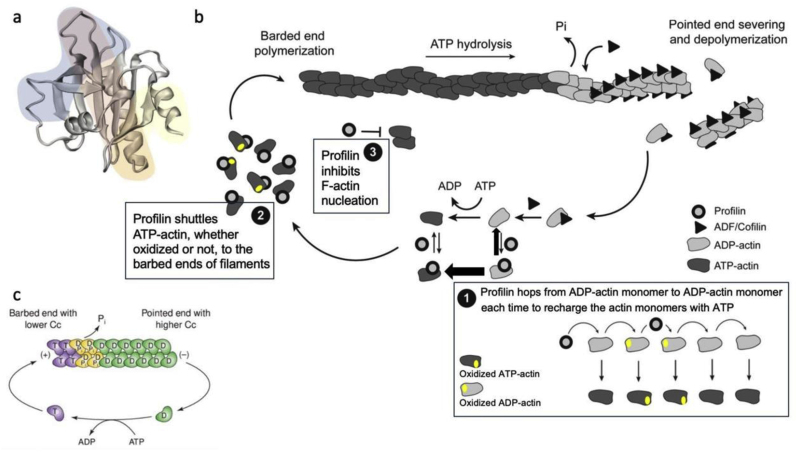
Roles of profilin and ADF/cofilin in actin treadmilling. Adapted from Pinto-Costa & Sousa (Fig. 2) [25]. (a) Structure of human profilin1 and its ligand binding domains: for actin (blue), PLP (yellow), and for PIP2 (red), adapted from Jockusch, Murk, and Rothkegel [26]. (b) In vitro, with purified protein, in the presence of salts, ATP, and antioxidants, actin can treadmill. This is the consequence of the difference in actin critical concentration at both ends. At the barbed end, the critical concentration of actin (0.1 μM) is ten times lower than at the pointed end (1 μM). Hence, at actin concentrations between 0.1 and 1.0 μM, monomers can add at the barbed end and detach at the pointed end. In addition, treadmilling of actin functions as an ATP triphosphatase, for each mole of actin incorporated in filaments, 1 mole of phosphate is being released, first attached to filaments then detached from filaments. (c) In vivo, G-actin concentration is well above the critical concentration of actin at both ends and thus filaments should elongate at both ends, but treadmilling can still occur, synergistically powered by profilin and ADF/cofilin proteins. At barbed ends, profilin (circles) enhances polymerization. Apart from its actin monomer sequestering activity which inhibits spontaneous nucleation of actin [box 3] to ensure selective actin polymerization driven by Wasp, Scar, Arp2 and Arp3 [27], profilin accelerates enzymatically ADP/ATP nucleotide exchange on G-actin [box 1] including actin monomers that have been moderately oxidized (gold oval dots), producing a pool of polymerizable (oxidized or not) actin subunits bound to ATP (dark gray G-actin). The profilin-G-actin (bound to ATP) complex can readily add to fast-growing-barbed ends, and thus profilin can shuttle G-actin to an F-actin barbed end then detaches from it [box 2]. ADF/cofilin loads cooperatively onto microfilament sides enriched of adenosine diphosphate (ADP)-actin (light gray G-actin) leading to F-actin severing and disassembly at pointed ends, hence the actin treadmilling observed in cells.

This is probably a key reason why G-actin interacts with profilin, an abundant protein in most cells. Profilin accelerates enzymatically (by up to 1000 folds) the exchange of the nucleotide bound to G-actin {Fig. 2} [[22], [23], [24], [25]]. Considering the vast excess of ATP over ADP (and AMP) in most cells, interaction of G-actin with profilin will result in G-actin bound to ATP {Fig. 2} due to the heightened off-rate of the ADP nucleotide from actin bound to profilin.

Importantly, it was shown that even limited oxidation of G-actin by ROS, when G-actin is bound to ADP blocks the ability of G-actin to polymerize, whereas moderately oxidized G-actin, once bound to ATP, retains intact ability to polymerize [24]. Hence, profilin is crucial to the reorganization of the cytoskeleton in cell exposed to agonists like growth factors, not only because of its ability to regulate signaling enzymes like phospholipase C-y1 but also because it maintains G-actin fully functional for participation in the cell cytoskeleton [24,28]. Since G-actin bound to profilin can bind to the barbed end of F-actin, which leads to the release of profilin and elongation of filaments, profilin contributes to the regulation of dynamic actin filaments [1,29].

Furthermore, once bound to ATP, G-actin can also bind thymosin beta 4 (Tβ_4_), a sequestering partner for actin which decreases the exchange of the nucleotide bound to actin (opposite effect of profilin) and thus, in the presence of profilin, sequesters polymerization-ready monomers as G-actin bound to ATP that can be released from Tβ_4_ to polymerize (due to the rapid off-rate of the actin-thymosin complex) [23]. And thus, in non-muscle cells, just about 65–85 % of actin is monomeric (in contrast to skeletal muscle where most actin is F-Actin), likely bound to G-actin binding proteins like profilin and Tβ_4_ [29]. Whether Tβ_4_ can also protect G-actin from excessive oxidation remains to be demonstrated.

**Fig. 3:**
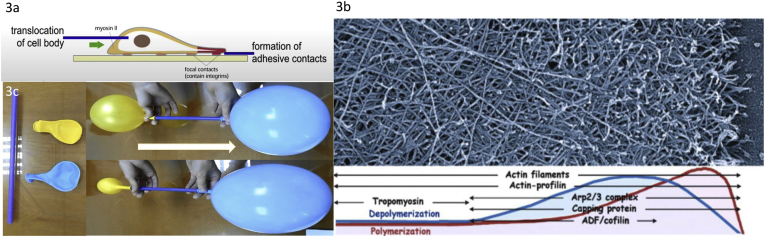

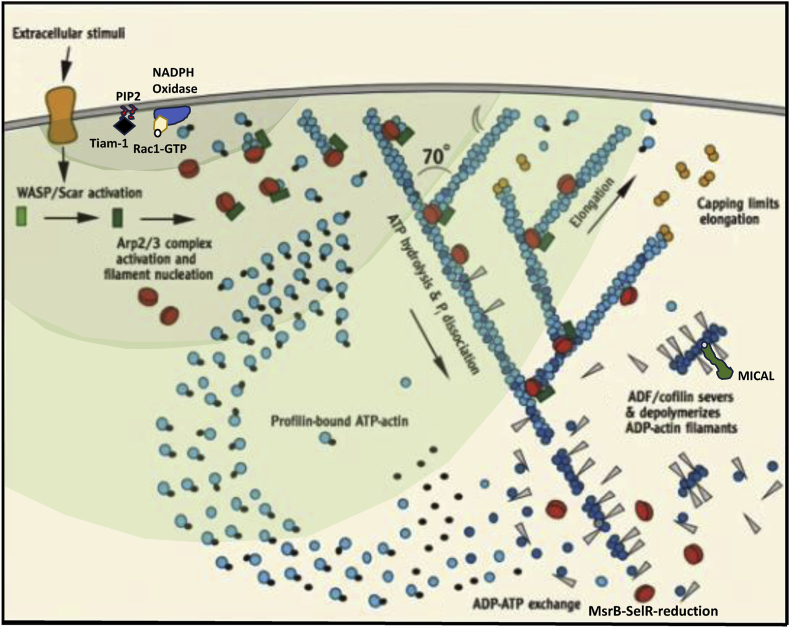
Actin treadmilling and the lamellipodia. (a) This first graph was copied from a video of Julie Theriot showing a fish keratinocyte in motion. Upon receiving extracellular signals like growth factors, many responsive cells polarize and develop a flattened expansion at one pole (lamellipodia), and a bulky pole at the opposite end containing the nucleus and other cellular organelles [31]. Such local flattening of the cell is mediated by criss-crossing actin filaments with anchors in both the ventral (attached to the substratum via focal adhesions) and dorsal plasma membrane, likely supported by myosin for contractility [3,30,31]. (b) Electron micrograph of a keratocyte provided by Tanya Svitkina and previously displayed by Pollard and Borisy [3], showing branched actin filaments at the very edge of the lamellipodia and longer actin filaments deeper within the lamellipodia. The diagram below shows the locations of key proteins [3]. The curves show regions of polymerization (red) and depolymerization (blue) of F-actin within the lamellipodia's advancing edge. (c) Such internal compression of the lamellipodia results in increased pressure at the advancing edge of the lamellipodia (considering Pierre Simon Laplace law and the cell plasma membrane as a complex bubble), P=C/r where P is the pressure inside a bubble, C in cells is a constant, and r is the radius of the bubble [35]. Hence, the smaller the bubble the greater the pressure inside the bubble. This continuously advancing actin compression machinery results in pressurizing the plasma membrane forward while stabilizing each advance through forward treadmilling and branching of actin filaments. Equilibrium of pressure across the cell is maintained by the cytoskeleton (possibly involving the wrinkles) [31] and the myosin mediated contraction at the rear end of the cell which detaches from the substrate as the cell advances. When two balloons of identical initial size when deflated, are inflated with one less than the other, upon connecting the balloons with a straw, the transfer of air is from the smaller balloon to the larger balloon, in a way that illustrates the bubble experiments of Laplace. (d) Molecular activities at the edge of the lamellipodia that support the compression machinery (figure adapted from the article of Thomas Pollard and Gary Borisy) [3]. Reorganization of the actin cytoskeleton involves the metabolism of membrane phosphatidylinositols, followed by activation of Rac1 through interaction with guanine nucleotide exchange factor Tiam-1 which is bound to phosphatidylinositol 4,5 bisphosphate and accelerates the binding of Rac1 to GTP. In turn, GTP-Rac1 activates NADPH oxidase (NOX1) with production of superoxide and ROS including H_2_O_2_ (green gradient in Fig. 3d), which contributes to the activation of WASP/Scar proteins. WASP/Scar bring together the Arp2/3 complex that nucleates new filaments and to form new branches of actin filaments at the edge of the lamellipodia. Such branching of filaments are necessary to generate the compression machinery of the lamellipodia at the edge. Capping proteins can limit the length of filaments, and filaments age by hydrolysis of ATP bound to each actin subunit of filaments (light blue to dark blue) followed by dissociation of the phosphate to complete the ATPase cycle of actin (see Fig. 3). ADF/cofilin promotes phosphate dissociation, severs ADP-actin filaments and promotes dissociation of ADP-actin from filament pointed ends. MICAL oxidation of actin filaments can accelerate markedly the depolymerization of filaments produced by ADP/cofilin. Profilin catalyzes the exchange of ADP for ATP (turning the subunits light blue again), thus returning actin subunits to the pool of ATP-actin bound to profilin, ready to elongate barbed ends as they become available. For MICAL oxidized G-actin, an additional step of reduction catalyzed by methionine sulfoxide reductase B1 (MsrB1) is required for G-actin to join the pool of ATP-actin bound to profilin and ready for polymerization.

In many instances for non-muscle cells, response to stimuli requires the swift transition of G-to F-actin and vice-versa [2,22,23] to sustain activities like protrusion and motion at the leading edge of a filopodium or a lamellipodium [3,30,31]. Local changes in cellular pressure at the very edge of cells may cause the initial membrane to bulge out, a protrusion that can be immediately consolidated by the polymerization of actin within the bulge with the support of a myriad of actin-binding proteins, and consequent reorganization of the cytoskeleton to produce motion {Fig. 3} [3,31,32]. The microtubular network and myosin are required for advancement of the cell body and the subsequent retraction of the cellular tail membrane [31,33,34]. The swift transition from G-actin to polymers at the advancing edge requires for the monomers to be bound to adenosine triphosphate (ATP) to elongate the barbed end, whereas dissociated monomers from filaments are mostly bound to adenosine diphosphate (ADP) {Fig. 3} [3]. The resulting treadmilling of actin filaments is required to advance the cell “compression machinery” that is required to maintain excess pressure at the advancing edge of cells {Fig. 3} [35].

## 3 ROS and reorganization of the actin cytoskeleton: impact of hypoxia and growth factors

The impact of ROS on actin is far from limited to denaturation and is now well recognized as a key mechanism for the actin cytoskeleton reorganization, both through the direct modification of actin, but also indirectly through the oxidation of actin-binding proteins, and other proteins that contribute to regulate the actin cytoskeleton [24,36]. Our initial lab observation at Johns Hopkins was that following a period of hypoxia, endothelial cells, upon reoxygenation, produce large concentrations of ROS, and concurrently undergo remarkable reorganization of their actin cytoskeleton, akin to those observed after addition of growth factors [36,37].

**Fig. 4:**
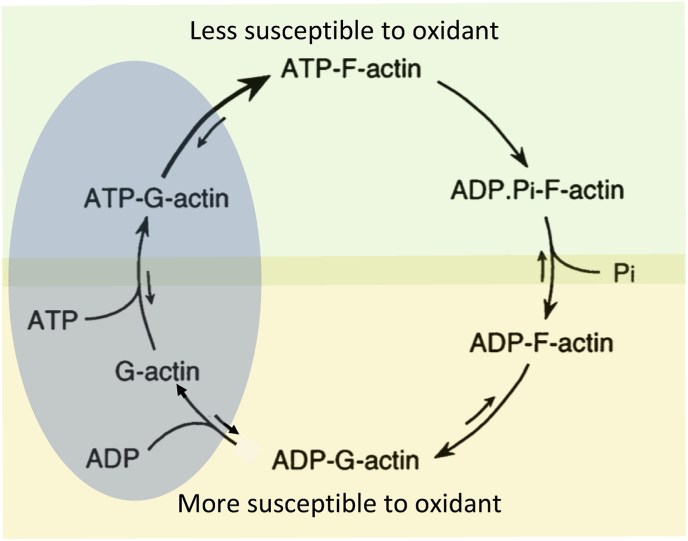
Nucleotide triphosphatase cycle of actin, and oxidation. G-actin is activated upon switching from the ADP-bound to the ATP-bound state. Actin binds ATP with a dissociation constant in the nanomolar range, well below the cellular ATP concentration. Cellular ATP concentration is always saturating, and thus physiological change in ATP concentrations doesn't regulate ATP binding to G-actin. Moreover, because ATP is always in large excess over ADP in cells, whenever ATP or ADP dissociates from G-actin it is nearly always replaced by an ATP. Spontaneous hydrolysis of G-actin bound ATP is usually slow, but ATP hydrolysis is 7000-folds faster when actin is polymerized (F-actin) than when it is monomeric (G-actin). ATP hydrolysis is the irreversible step in the actin cycle, forcing actin to pass through an ADP-bound intermediate state. ADP actin is more susceptible to oxidation, and oxidized ADP-G-actin is no longer polymerizable, whereas oxidized ATP-actin retains its ability to polymerize [24]. Hence, the impact of profilin on the replacement of ADP for ATP for oxidized G-actin is critical to physiological actin responses. The green and orange shades contain the conformations of actin that are less or more susceptible to oxidation, respectively. The blue oval shade indicates the conformations impacted by profilin.

This observation was the first time we realized that oxidants like superoxide and derived ROS could signal the actin cytoskeleton to reorganize [36,37]. At the time of this discovery, the concept that oxidants could contribute to actin's biology was not always warmly looked upon by cell biologists. Instructively, Rac1, a protein that belongs to the large Ras and Rho family of small GTPases, functions as a molecular switch, cycling between active (GTP-bound) and inactive (GDP-bound) states [38]. Rac1 was known to play a crucial role in regulating various cellular processes, including actin cytoskeleton dynamics that cause membrane ruffling, cell migration, and cell cycle progression {Fig. 3} [39]. Hence activated Rac1 (bound to GTP or V12-mutated) controls actin's interaction with its own filaments in a way that is impacted by the adenosine nucleotide bound to G-actin (ATP versus ADP) {Fig. 3}, and also impacted by its oxidation status and thus polymerizability {Fig. 4}.

In neutrophils, Rac2 is responsible for activating the NADPH oxidase complex, which generates superoxide and downstream ROS. We hypothesized that these two functions of Rac, activations of the actin cytoskeleton and of NADPH oxidase enzymes, could be connected [37]. Nowadays, it is well recognized that post-translational modification of actin, and of actin binding proteins, including through oxidation, methylation (nuclear actin), arginylation, nitration, phosphorylation, S-nitrosylation, S-glutathionylation, and acetylation, is critical to allow actin and its network to adapt to rapidly changing biological environments [10].

Coincident with our work on the impact of ROS on the actin cytoskeleton, the team of Toren Finkel at the NIH showed that cellular responses to PDGF: tyrosine phosphorylation, DNA synthesis, motility, etc., were suppressed when the growth factor-stimulated rise in hydrogen peroxide (H_2_O_2_) concentration was blocked [18]. Deactivation of protein tyrosine phosphatases (PTP) through selective oxidation is necessary to allow tyrosine kinases to phosphorylate and thus activate or deactivate their substrates effectively [40]. Such targeted oxidation and that of other critical signaling proteins is likely responsible for the impact of PDGF on cells [18,38,40]. Indeed, limited oxidation of actin and its binding proteins is also believed to account for some of the effects of ROS on the cytoskeleton [5,24,36,37,41,42].

## 4 Superoxide, ROS and the oncogenic transformation of cells

Our team showed that transformation of fibroblasts (NIH3T3 cells) with HRas^V12^ resulted in increased production of superoxide and derived ROS that was mediated by Rac1 [43]. This was the first time that the transformation of cells by an activated oncogene was shown to require superoxide and derived ROS overproduction. Indeed, we showed that the mitogenic activity of these transformed fibroblasts needed the production of superoxide by a flavoprotein activated by Rac1. The team of Griendling and Lambeth discovered the specific Rac1-activated flavoprotein: NAD(P)H oxidase 1 (MOX1, which was then renamed NOX1). NOX1 is responsible for the production of superoxide in many non-phagocytic cells [44]. NOX1 was later shown to be responsible for superoxide generation and oncogenic transformation of fibroblasts, a finding consistent with our original discovery [45].

**Fig. 5:**
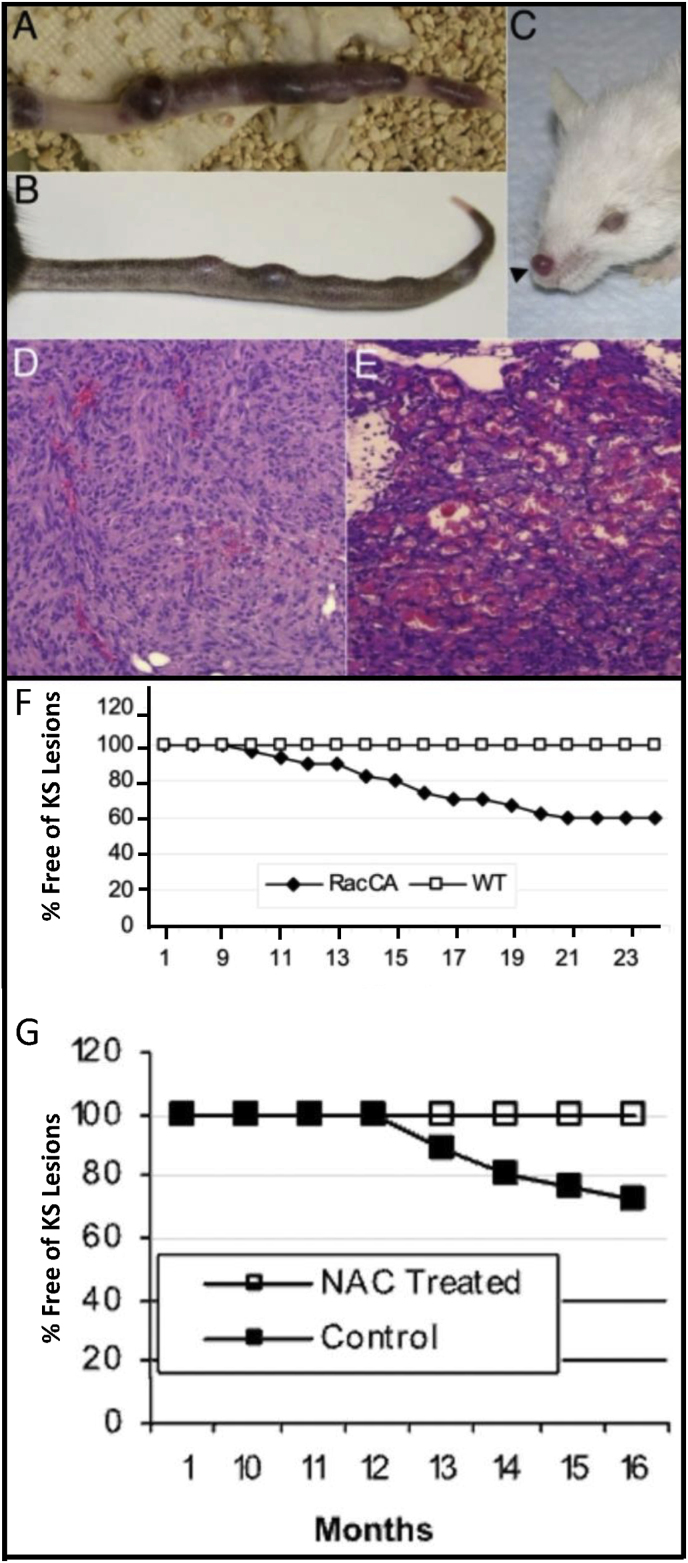
Dependence of KS tumorigenesis on Rac 1 and ROS. Adapted from Ma et al. [46] KS-like tumors on the tail (a and b) and nose (c) of mice expressing a constitutively active Rac1^V12^ (RacCA^+/+^). H&E staining of early (d) and late stage (e) neoplastic tissue of RacCA^+/+^ mice showing pathologic features identical to that of human KS. The necessary presence of RacCA^+/+^ for the development of KS is shown in (f). The dependence on ROS for KS generation in RacCA^+/+^ mice is demonstrated by the impact of N-acetyl-l-cysteine (NAC) on tumor-free survival of homozygous male RacCA^+/+^ mice (g).

Next, we created a mouse model that replicated identically the human Kaposi's sarcoma (KS) {Fig. 5}. KS is the most frequent cancer associated with AIDS, and in our mouse model, was induced by overexpression of a constitutively activated Rac1 (Rac1^V12^) using the smooth muscle cell actin promoter for expression (likely resulting in Rac^V12^ pericytes) [46]. This model was indistinguishable from the classic type of KS, not only with its purple-brown skin lesions that were only seen in skin exposed to light (nose, ears, paws, and tail), but also because of the 10:1 male to female ratio, and the higher prevalence of KS lesions in aging mice (greater than nine month old). We further demonstrated that the KS tumors formed only in the presence of superoxide and derived ROS, as we could suppress completely the development of KS lesions by scavenging ROS with *N*-acetylcysteine in our transgenic Rac^V12^ mice (RacCA^+/+^).

Cancer cells must proliferate and migrate to invade tissue, surrounding tissues like lymph nodes, and distant tissues, as in metastases. This is not unique to cancer cells as many immune cells, neutrophils, and macrophages, for example, are capable of growth, migration, and invasion of distant tissues [47,48]. What is rather unique to cancer cells is the intensity of their metabolic activity. For instance, we detect cancer cells based on this intensity by using a positron emission tomography (PET) scan–an imaging test that can help reveal the metabolic activity of our tissues using a tracer called fluorodeoxyglucose (FDG). This radioactive sugar is readily absorbed by cancer cells, with their high metabolic rate. Hence, cancer cells on a PET scan appear as "hot spots" [49].

The accelerated metabolic activity of cancer cells requires a unique supply chain of calories and can depend on an actin cytoskeleton-supported membrane activity called membrane ruffling. Membrane ruffling is the Rac1-dependent dynamic protrusions of the cell membrane and fluid-phase pinocytosis elegantly imaged by Eric Betzig and his team [50].

Cellular injection of H-Ras in quiescent fibroblasts can induce membrane ruffling and fluid phase pinocytosis [51]. Such ruffling activity results in the engulfment of extracellular fluid and exosomes produced by surrounding tissues inside cancer cell vesicles [52,53]. Membrane ruffling is the fastest way for cells to accumulate glucose and other energy sources, as well as other cellular exosome components [54] like lipids, proteins, carbohydrates, and microRNAs contained in exosomes, that are necessary to build tumors [[50], [51], [52], [53], [54]]. Ruffling is not unique to cancer cells but is typically found in cells with accelerated metabolism like the placenta at the maternal/fetal interface [55], or in primary innate immune cells, most often immature dendritic cells and macrophages that can internalize their entire cell surface every 33 min [56,57].

Rac1 activation by H-ras leads to ROS production by NOX1. ROS activates Src, and the major target of Src is cortactin [58,59]. Phosphorylation of cortactin, particularly its tyrosine phosphorylation by Src, is strongly correlated with increased cortactin-induced ruffling activity and enhanced migration and invasiveness of cancer cells. Cortactin seems to mediate these cancer cells activities via ARP2/3 activation [60].

Hence, the higher production of ROS and NOS triggered by Rac1 activation allows for PTM of actin and actin-binding proteins, including oxidation, S-nitrosylation, S-glutathionylation, phosphorylation, and arginylation, that are essential for motility. Rac1-mediated increases in ROS have been shown to affect actin dynamics, as has ROS-dependent actin glutathionylation (a post translational modification to actin's cysteine residues) [37,[61], [62], [63], [64], [65], [66]]. Arginylation of actin is actively being investigated as it may involve zipcode-mediated actin mRNA targeting to the cell periphery, proposed to mediate local synthesis of actin at the cell leading edge. N-terminal arginylation specifically targets non-muscle β-actin via a nucleotide-dependent mechanism, and has been observed in cancer cells [67].

**Fig. 6:**
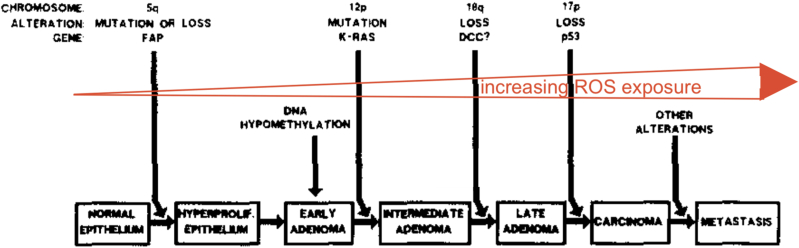
Increasing ROS production and tumor progression: the colorectal cancer example. Adapted from Fearon and Vogelstein [72]. As we have shown, oncogenes like HRas^V12^ can induce ROS production which can activate signaling pathways that promote cell proliferation. Further elevation of ROS levels can induce DNA mutations, leading to genomic instability—a hallmark of cancer cells as they support the development of carcinoma and metastasis.

The link between ROS and cancers is now well established, early tumor formation requires limited generation of ROS to expand, and later in tumors, larger concentrations or ROS lead to more frequent mutations and cellular apoptosis {Fig. 6} [68,69]. Recently, the bacterium Helicobacter (H) Pylori, through a FabX [4Fe–4S] cluster that is essential to the synthesis of unsaturated fatty acids, was shown to produce superoxide [70]. Such superoxide production could be critical to the development of gastric cancer induced by H Pylori, and the migration and metastases of gastric cancer cells to several organs [71]. Interestingly, redox mediated glutathionylation of actin has also been demonstrated to affect cancer cell migration and invasion [62]. In summary, the simple observation that an oncogene like HRas^V12^ could trigger the production of ROS via Rac1 and NADPH oxidase to transform cells has lead to a better understanding of the initiation and progression of cancers [71].

## 5 ROS and tissue repair, actin mediated motility of repair cells

**Fig. 7:**
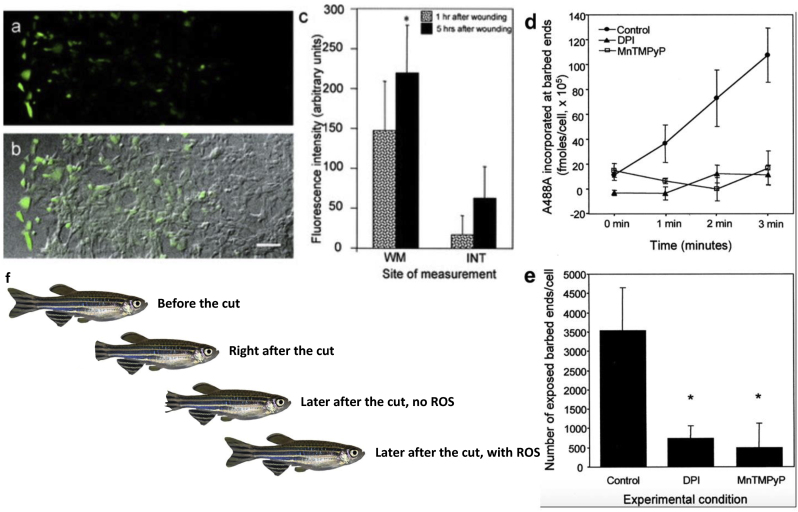
ROS, cell motility, and wound healing. Adapted from Moldovan et al. [42] Confluent monolayers of endothelial cells (EC) were scratched with a glass tip to create a wound. The generation of intracellular ROS was monitored by preloading the cells with CM-DCF-DA that fluoresces upon exposure to oxidants, especially H_2_O_2_. Cells before scratching or cells distant from the scratched area displayed very low CM-DCF-DA fluorescence **(a–c)**. Differential interference contrast (DIC) image and fluorescence image of the same field were overlaid (b). EC flanking the wound (wound margin or WM) were consistently and significantly more fluorescent than more distant cells (intact monolayer or INT) when observed at 1 and 5 h (c). Bar in lower right corner = 100 μm. Furthermore, migration of EC into the “empty” wound space was shown to be dependent on the presence of ROS (see reference 42 for **videos** of cell motility with and without ROS). Actin polymerization was accelerated in EC with increased production of ROS, specifically H_2_O_2_. Thus, when ROS production was inhibited by DPI or MnTMPyP, migration of EC was suppressed markedly and polymerization of actin measured by incorporation of fluorescent actin (A488A) into filaments was inhibited **(d)**, possibly due to a limiting number of barbed ends for monomers to be added **(e)** [13,42]. Since WASP requires PIP2 for activation and because ROS increase PIP2 (increased synthesis, decreased activity of phosphatases) antioxidants might reduce available barbed ends by preventing WASP activation. Representation showing four zebrafish: Before (top) and after (next three) partial caudal fin amputation, right after amputation (second from top), then later after amputation, either in conditions that block H_2_O_2_ (cut and no ROS, third from top), or healing without inhibition of H_2_O_2_ (cut with ROS, fourth from top). Regeneration of the Zebrafish's caudal fin has been shown to be dependent on release of H_2_O_2_ along the wound margin to block phosphatases. When this initial burst of H_2_O_2_ is inhibited post wounding (cut and no ROS), regeneration of the caudal fin does not occur **(f)**.

While ROS are essential for early tumor formation and progression, they are also required for tissue repair and regeneration [11,12,42,71,72]. We observed that, following injury of an endothelial monolayer (scratch assay), the cells adjacent to the injury site were induced immediately to produce ROS and in particular hydrogen peroxide (H_2_O_2_) on each side of the wound {Fig. 7} [42]. This production of ROS was about fifteen to twenty cell rows deep in the monolayer. H_2_O_2_ production was followed by the migration and proliferation of endothelial cells on the edge of the wound to cover the empty space and restore an intact endothelial layer, without the addition of any growth factors [13,42].

Inhibition of ROS reduced markedly the migration and proliferation of endothelial cells, and consequently, repair of the wound, thus further suggesting that ROS are necessary for motility supported by the actin cytoskeleton [42]. Overexpression of Rac1^v12^, the constitutively activated mutant of Rac1, increased the concentration of F-actin and induced reorganization of the actin cytoskeleton with more F-actin in membrane ruffles of endothelial cells, and such increase and changes were prevented by the exposure of these cells to antioxidants [13,42].

Soon after these observations, the laboratory of Chandan K. Sen reported *in vitro* evidence that H_2_O_2_ supports wound healing by inducing vascular endothelial growth factor (VEGF) expression in human keratinocytes [14]. Sen's team provided *in vivo* evidence, that the redox environment of a wound site can affect healing outcomes. Indeed, low concentrations of H_2_O_2_ support the healing process, while higher concentrations of H_2_O_2_ prevented healing.

More recently, the team of Jeroen den Hertog showed that amputation of the caudal fin of zebra fish also results in rapid and transient increase in H_2_O_2_ at the wound margin {Fig. 7f}, which is essential for fin regeneration [11]. Inhibition of ROS blocked regeneration of the fin, as ROS must induce the oxidation of the catalytic site of protein tyrosine phosphatases (PTP) to reduce their activity [73]. More specifically, in another publication, of the eight PTP shown to undergo significant increases in oxidation post-amputation, Shp2 oxidation was required for Xenopus tadpole tail regeneration [12]. As mentioned earlier, membrane ruffling induced fluid-phase macropinocytosis is an essential process to capture not only extracellular fluid but also small vesicles (like exosomes) likely to contribute to processes of tissue repair described above, but also organogenesis of the fetus during pregnancy, and tumor formation [35,39,51,[74], [75], [76]].

## 6 Inflammation, repair, and aging

While oxidation is only second to phosphorylation in terms of the number of actin residues that can be modified, nitration, together with S-nitrosylation, come third [77] {Table 1}. Next, we describe how S-nitrosylation and S-glutathionylation can impact key aspects of inflammation.

**Table 1:** Specific PTMs of actin; adapted from Terman & Kashina 2012 (Table 1) [77].

Modification	Known Sites on Actin (∗ = known functionally important modification)
Acetylation	Met-1∗, Asp/Glu-2∗, Asp-3∗, Lys-50, Lys-61, Lys-68, Lys-191, Lys-326, Lys-328
ADP-Ribosylation	Arg-28, Arg-95∗, Thr-148∗, Arg-177∗, Arg-206∗, Arg-372∗
Arginylation	Asp-3∗, Ser-52, Ile-87, Phe-90, Gly-152, Leu-295, Asn-299
Carbonylation	His-40, His-87, His-173, Cys-374∗
Crosslinking	Lys-50 (with Glu-270)∗
Glutathionylation	Cys-217, Cys-374∗
Isoaspartylation	Asp-25, Asp-179
Malonylation	Lys-61
Methylation	His-73∗, Ile-87, Asn-299, Lys-326
Nitration	Tyr-53, Tyr-69∗, Tyr-91, Tyr-198, Tyr-218, Tyr-240, Tyr-294, Tyr-362
Nitrosylation	Cys-217, Cys-257, Cys-285, Cys-374∗
O-GlcNAcylation	Ser-52, Ser-155, Ser-199, Ser-232, Ser-323, Ser-368
Oxidation	Met-44∗, Met-47, Trp-79, Met-82, Trp-86, Met-178, Met-190, Cys-217, Met-227, Cys-257, Met-269, Cys-272∗, Cys-285∗, Met-325, Trp-340, Met- 355, Trp-356, Cys-374∗
Phosphorylation	Ser-14, Ser-33, Ser-52, Tyr-53∗, Ser-60, Thr-66, Tyr-69, Thr-89, Tyr-91, Thr-148, Thr-160, Thr-162, Tyr-166, Tyr-169, Thr-186, Tyr-188, Tyr-198, Ser-199, Thr/Ser-201∗, Thr-202∗, Thr-203∗, Tyr-218∗, Thr-229, Ser- 233, Ser-239, Tyr-240, Thr-262, Tyr-294, Thr-297, Tyr-306, Thr-318, Ser- 323, Ser-324, Tyr-362, Ser-365
SUMOylation	Lys-68∗, Lys-284∗
Transglutamination	Gln-41∗
Ubiquitylation	Lys-50, Lys-61, Lys-68, Lys-84, Lys-113, Lys-118, Lys-191, Lys-291, Lys- 315, Lys-326, Lys-328

Neutrophils play a key role in immune responses, and integrin adhesion is vital for their activation and recruitment to sites of inflammation through their adhesion to endothelial cells [78,79]. The complex interaction of integrins with actin has been shown to be critical for the adhesion of neutrophils [80]. The laboratory of Tatyana Milovanova, with their work on the impact of hyperoxia and redox conditions on neutrophil β_2_ integrin adhesion, underscored the importance of these redox reactions. This work supports that excessive S-nitrosylation of actin is critical for the inhibition of neutrophil β_2_ integrin adhesion under hyperoxic conditions [78].

Under hyperoxic conditions, such as hyperbaric oxygen used by Thom et al., ROS (e.g., superoxide and hydrogen peroxide) and RNS (e.g., nitric oxide and nitrogen dioxide) are concurrently produced in neutrophils. The production of nitric oxide, NO, by nitric oxide synthase, NOS, results in S-nitrosylation of actin at the cysteine residues near the carboxyl-terminal end. Actin S-nitrosylation disrupts the intracellular distribution of actin in neutrophils, thereby inhibiting the clustering of β_2_ integrins and impairing neutrophil adhesion mediated by β_2_ integrins. The inhibitory effect of hyperoxia on integrin function could be prevented by l-NAME (N^8^-nitro-l-arginine methyl ester, a non-selective inhibitor of NOS) and could be reversed by exposing neutrophils to ultraviolet light, which de-nitrosylates actin. Conditions of neutrophil adhesion and inflammation, such as ischemia-reperfusion injury, smoke-induced lung injury, and carbon monoxide poisoning, can be impacted by hyperbaric oxygen, reducing inflammation associated with these conditions [78,79].

The same team focused on Vasodilator-stimulated protein (Ena/VASP). VASP is a multifunctional organizer of the actin cytoskeleton that can nucleate and bundle actin filaments. VASP exhibits a high affinity for S-nitrosylated short filaments of actin, which promotes actin polymerization through the increased concentration of barbed ends. VASP-assembled actin barbed-end bundles also include Rac1, cyclic AMP-dependent, and cyclic GMP-dependent protein kinases [78,79,81].

Reducing cell concentrations of any of these proteins with small inhibitory RNA prevented the enhanced free barbed ends’ formation, increased actin polymerization, and integrin inhibition by hyperoxia. Activation of either cyclic AMP-dependent or cyclic GMP-dependent protein kinase, caused the phosphorylation of VASP on serine 153. Phosphorylation of VASP blocked the polymerization of S-nitrosylated actin, VASP binding to actin, increased Rac activity, and elevated formation of actin free barbed ends, and restored normal β_2_ integrin function [78,79,81].

It is instructive to observe the reversibility of changes in the actin cytoskeleton induced by RNS and ROS. At low ROS concentrations, oxidation of cysteine and methionine residues (as in the case of MICAL) can be reversed by redox proteins (like methionine sulfoxide reductases). Such reactions affect interactions of actin with self and with binding proteins and thus modify actin structures dramatically. These are the redox reactions that are seen, for example, early on during tumor formation [10,[82], [83], [84]].

Another key redox mediated post translational modification (PTM) of actin is S-glutathionylation, which precisely targets cysteine residues (C374 and C217) [10,61,85,86]. Triggered under oxidative conditions–such as ROS generation, nitrosative stress, and ischemia-reperfusion–this PTM consists of the reversible linkage of glutathione to a cysteine's thiol side chain, forming a mixed disulfide [10,[63], [64], [65], [66],^87^,^88^]. This process is subject to enzymatic regulation, with glutathione S-transferase π (GSTπ) capable of catalyzing the reaction and Glyoxalase II potentially facilitating S-glutathionylation [89,90]. Instructively, Kalinina & Novichkova described the capability of glutaredoxins (Grx) to catalyze not only de-glutathionylation, but also promote s-glutathionylation depending on the cellular GSH/GSSG (reduced glutathione/oxidized glutathione) ratio [88].

This modification significantly affects actin's behavior in several ways. Regarding polymerization, glutathionylated G-actin shows reduced polymerization efficiency, while de-glutathionylation can increase polymerization rate by up to 6-fold [61,[63], [64], [65]]. S-glutathionylation also affects actin's interactions. For example, it can lead to reduced binding cooperativity with tropomyosin and diminished actomyosin-S1 ATPase activity [64,91]. Additional to regulatory effects on actin polymerization, the reversible glutathionylation of cysteine's thiol side chains can protect actin from irreversible oxidation (e.g. oxidation to sulfinic or sulfonic acid) under oxidatively stressful conditions [66,88]. This PTM can also target actin binding proteins, such as cofilin, reducing their ability to depolymerize F-actin [66].

These molecular changes have significant implications at the cellular level. The modification impairs cell spreading and cytoskeletal organization and disrupts neutrophil function [63,86,92]. Through these various effects, actin glutathionylation serves as a critical regulatory mechanism for actin dynamics, influencing both normal cellular function and disease states.

Our renewed understanding of the dual impact of ROS on the biology of cells and tissues supports that low concentrations of ROS promote tissue repair, or tumors in cells that show a proclivity for cancer transformation. Whereas higher concentrations of ROS can lead to apoptosis, necrosis, and consequent tissue damage, or to more aggressive cancers. Such concepts helped us understand better widespread pathological conditions like cancer and atherosclerosis [68,69,93,94].

**Fig. 8:**
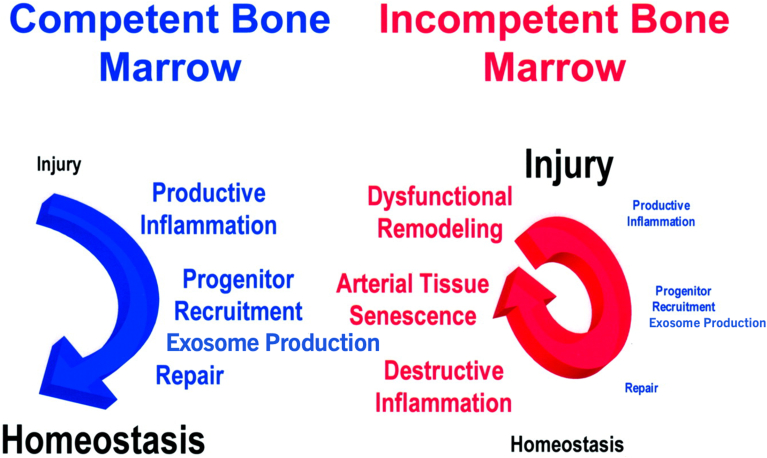
Inflammation, a ROS-dependent process, can drive tissue repair or destruction. Adapted from Goldschmidt-Clermont et al. (Fig. 2) [95]. Atherosclerosis, an inflammatory process that leads to the obstruction of arterial conduits, remains the major cause of death and morbidity. Arterial injuries due to smoking, high blood pressure, diabetes mellitus, or elevated blood lipids, and other environmental stressors, happen daily. There is a mechanistic link between arterial repair and inflammation. When a competent bone marrow is present, the inflammatory reaction following arterial injuries is self-limited and constructive by triggering the circulation of progenitor cells and the production and release of exosomes produced by these cells which promote tissue repair and immune modulation (arterial homeostasis). Indeed, vascular progenitor cells that are capable of arterial repair can egress the bone marrow and participate in repair processes that are highly dependent of Rac1 and Rac2, and thus, on the production of ROS [101]. The self-limited nature of this productive inflammatory reaction is due to the fact that arterial wall repair leads to the cessation of the signal triggering the inflammatory response. But as we become older, age becomes the dominant risk for atherosclerosis [96]. It is highly likely that loss of competent progenitor cells in the bone marrow capable of repair with aging contributes significantly to the aging risk [100]. Hence, in the presence of incompetent bone marrow, there is a lack of arterial repair which perpetuates, and potentially, exacerbates atherosclerotic inflammation. This yields a destructive positive feedback loop, with progressive senescence and dysfunction of the arterial wall, arterial wall remodeling with atherosclerotic plaques whose rupture leads to myocardial infarction, stroke, and peripheral arterial occlusion (lack of arterial homeostasis).

As it relates to inflammation, a similar impact of inflammation can be considered where low levels of inflammation are designed to trigger tissue repair, or even regeneration as in tadpoles, salamanders, or zebrafish, whereas higher levels of inflammation can lead to the destruction of tissues [95]. Atherosclerosis is the one condition most frequently associated with morbidity and mortality (myocardial infarctions, strokes, sudden cardiac death) [95,96]. Many risk factors like smoking, hypertension, hyperlipidemia, family history of arterial conditions, contribute to atherosclerosis. However, as we age, aging itself becomes the dominant risk for cardiovascular events, a risk that is not explained by worsening risk factors like smoking, elevated blood lipids or high blood pressure {Fig. 8} [[95], [96], [97], [98]].

Instead, the greatest impact of aging on atherosclerosis is the progressive failure of repair capacity [99,100]. As mentioned, repair is triggered by low levels of inflammation. But when repair does not ensue, the negative feedback loop that subsides inflammation fails to occur [95,99,100]. Consequently, inflammation worsen to the point of causing and/or accelerating atherosclerosis with steps like endothelial dysfunction, proliferation and migration of smooth muscle cells to arterial plaques, and colonization of the arterial wall by inflammatory cells. And all these steps have been connected to the production of ROS [[101], [102], [103], [104], [105]].

## 7 Actin, speed of cell response, ROS and MICAL

We do not yet fully comprehend the details of how ROS (like superoxide, H_2_O_2_, and hypochlorous acid) regulate activities of the actin cytoskeleton that are required for inflammation to expand and destroy tissues. However, what we do know is that rapid responses of the actin cytoskeleton in cells do require “enzymatic-like” speed of reactions and there are many examples of such:

(1) the enzymatic speed of exchange of the nucleotide bound to monomeric actin promoted by profilin with or without the presence of Tβ_4_ [23].
(2) the transition from gel to solution of actin filaments in the presence of gelsolin [106].
(3) the disassembly of actin filaments, either individual or bundled filaments, in the presence of activated MICAL enzymes, to name just a few [107,108].

MICAL proteins are flavoprotein monooxygenase enzymes that use nicotinamide adenine dinucleotide phosphate (NADPH) to catalyze oxidation-reduction (redox) reactions [5,10,107]. MICAL binds F-actin and directly converts Met44 (highly conserved residue) to MetO44, which reorients the side chain of M44 and induces a new intermolecular interaction of actin residue M47 (M47-*O*-T351). This new interaction disassembles F-actin and alters actin polymerization {Fig. 3d} [3,10]. The actin loop that contains M44 is located at the pointed-end of one actin monomer faced by the barbed-end of the adjacent actin monomer within the filament.

Oxidation of Met44 is believed to be the mechanism responsible for the collapse of filaments induced by MICAL, although the activity of severing proteins like ADP/cofilin could also be exacerbated by the presence of MetO44 [10]. MICAL-oxidized actin can undergo extremely fast (84 subunits/s) disassembly, which depends on Factin's nucleotide-bound state [107]. But in conjunction with ADP/cofilin, MICAL oxidation of actin promotes F-actin disassembly independent of the nucleotide-bound state. The oxidation of actin by MICAL can be reversed by methionine sulfoxide reductase B SelR (MsrB-SelR) {Fig. 3d}, which reduces the oxidized methionine residues and restores actin's normal structure and function [108]. Reduction of oxidized methionine also restores profilin's ability to promote actin repolymerization. This reversible redox regulation allows for dynamic control of actin filaments in response to cellular signals and resulting ROS production.

## 8 Oxidation of the actin cytoskeleton: superoxide versus nitric oxide

Often in cells, ROS are being produced concurrently with reactive nitrogen species (RNS) like nitric oxide (NO), and their respective effects on the actin cytoskeleton may antagonize or synergize depending on surrounding conditions and the specific proteins they target [5,10,109]. NO, which is enzymatically generated by nitric oxide synthase (NOS), S-nitrosylates Cys374 of actin [5,10]. This modification blocks interaction of actin with profilin, resulting in reduced production of actin structures in response to agonists [10]. Hence, both NO and superoxide can affect the actin cytoskeleton in a way that is targeted and specific. At low ROS concentrations, oxidation of cysteine and methionine residues (as in the case of MICAL) can be reversed by redox proteins (like methionine sulfoxide reductases). Such reactions affect interactions of actin with self and with binding proteins and thus modify dramatically actin structures. These are the redox reactions that are seen for example early on during tumor formation [82].

More intense oxidative stress promotes disulfide bridges or disulfide bonds with glutathione and can lead to irreversible alteration of protein structures where cysteine and methionine residues can become irreversibly modified (sulfinic and sulfonic acid, and methionine sulfone) leading to apoptosis or necrosis of cells, as seen in more advanced tumors for example [110].

## 9 Redox-driven actin modifications: mechanisms, disease, and therapeutic opportunities

Findings from recent developments in the field of actin post translational modifications (PTMs) reveal several important patterns in our understanding of redox-mediated modifications of actin and associated proteins, their role in disease processes, and therapeutic potential.

The molecular mechanisms underlying these modifications appear to be highly conserved and specific. MICAL enzymes demonstrate precise targeting of methionine residues (M44 and M47) on actin, leading to precipitated ADP-bound F-actin disassembly [107]. Indeed, this process occurs at a remarkable rate of 84 subunits per second, suggesting its potential as an instant response to cellular stress with depolymerization of F-actin near the pointed ends of filaments. The interaction between MICAL-mediated oxidation and cofilin activity appears particularly significant, with evidence showing that MICAL1 oxidation enhances cofilin-mediated severing and bypasses normal regulatory mechanisms [111]. Hence, it can be expected that the dissociation of MICAL-mediated F-actin oxidation from cofilin activity could impact cellular processes.

In disease contexts, these modifications show distinct patterns. In cancer, altered MICAL expression, particularly MICAL1 and MICAL2, appears to play a crucial role in disease progression and metastasis [83,84]. The finding that MICAL regulation can sensitize cancer cells to existing treatments like Gleevec suggests potential therapeutic applications through combination approaches [82]. Furthermore, dissociation of MICAL-mediated F-actin oxidation (M44 and M47) from cofilin activity could be effected using small molecules or peptides that specifically block MICAL's activity, or with cofilin-specific inhibitors such as Cofilin-Tat peptides.

Tauopathies are a group of neurodegenerative diseases characterized by the abnormal accumulation and aggregation of the Tau protein in the brain, leading to neurofibrillary tangles and neuronal dysfunction, as seen in Alzheimer's Disease. The formation of cofilin-actin rods under oxidative stress conditions appears to be a key pathological mechanism for Tauopathies, as these rods can sequester phosphorylated tau, providing an important link between cytoskeletal dysfunction and classic hallmarks of neurodegeneration [112,113]. And here again, the dissociation of MICAL and cofilin activities could potentially slow down, or reverse, the advancement of tauopathies.

The therapeutic implications of these findings are significant. The identification of multiple targetable pathways, particularly those involving MICAL enzymes and their regulatory networks, suggests several potential intervention points. The finding that PAK1 phosphorylation of MICAL1 enhances its actin-disassembly activity points to the possibility of modulating these pathways through existing kinase inhibitors [114].

In amyotrophic lateral sclerosis (ALS), mutations of the gene of profilin (PFN1), the actin-binding protein that accelerates enzymatically the actin nucleotide exchange, have been identified as one of the genetic causes for the disease [23]. PFN1 mutations in ALS are typically inherited in an autosomal dominant manner, as the presence of the mutation in one allele is enough to cause the disease. The most studied mutations in PFN1 in ALS occur at specific residues, and particularly a mutation at position H119 of profilin. H119 is critical to the interaction of profilin with actin and to the impact of profilin on nucleotide exchange (it is the residue of profilin closest to the nucleotide cleft of actin) [115]. We have shown that Schwann cells extracellular vesicles (SCEV) obtained from healthy donors and injected intravenously to a patient with advanced ALS seemed to be able to stabilize the progression of ALS, at least for a while [116]. Hence, SCEV may be able to deliver their cargo to cells (Schwann cells, neurons, muscle cells) involved in the progression of ALS. If that is the case, and profilin was shown to be essential for the biology of Schwann cells, then injections of SCEV from normal donors (containing unmutated profilin 1) could prevent the development/progression of ALS [117]. The same could be true for other genes that, when mutated, can cause ALS (like SOD1, etc.)

However, several important questions remain unanswered. While the molecular mechanisms are well-characterized *in vitro*, their behavior in complex cellular environments, particularly during disease progression, requires further investigation. The temporal dynamics of these modifications and their role in disease onset versus progression remain unclear. Additionally, while therapeutic targeting shows promise, the potential off-target effects of modifying such fundamental cellular processes need careful consideration.

## 10 Conclusion

Hence, after 30 years of hard work by an army of laboratories, we have come to realize that redox activity is not simply a trigger for cellular noxious reactions, like the denaturation of G-actin after purification, but instead can induce carefully choreographed and essential processes for life, and including the genesis of organs in the fetus, protection against infectious agents and repair of damaged tissues following injuries. Such advances were difficult to anticipate thirty years ago when it was unconventional to fathom that ROS could have such beneficial impact for cells and tissues. We hope that this review will trigger new curiosity and discoveries about the actin cytoskeleton, cell motility, and the impact of ROS on actin biology, as well as in human health and illnesses {Table 2}.

**Table 2:** Suggestions for future research on actin and REDOX.

Areas of Research	Possible Direction(s)
Novel ROS-responsive actin regulators	● Identify novel redox-sensitive actin-binding proteins through proteomic approaches. Characterize their structural and functional changes upon oxidative modifications. ● Investigate the interplay between MICAL-mediated actin oxidation and cofilin activity, exploring dissociation strategies that could impact cellular processes. ● Examine PAK1 phosphorylation of MICAL1 as a regulatory mechanism and its potential for therapeutic intervention.
Therapeutic ROS modulation	● Develop targeted antioxidant delivery systems to modulate ROS precisely in pathological settings (e.g., cancer, chronic wounds). ● Investigate small molecules or biologics that can selectively enhance or inhibit ROS effects on actin dynamics. ● Explore small molecules or peptides that specifically inhibit MICAL activity or disrupt MICAL-cofilin interactions. - Assess combination approaches targeting MICAL enzymes for sensitizing cancer cells to existing treatments, such as Gleevec.
Extracellular vesicles (EVs) and actin remodeling	● Explore how ROS-driven actin reorganization affects EV release and uptake. ● Assess EVs as carriers of redox signals and their impact on tissue repair or cancer metastasis. ● Investigate Schwann cell-derived extracellular vesicles (SCEV) as potential therapeutic agents for ALS, focusing on their ability to deliver functional profilin and other key cytoskeletal regulators.
ROS and actin in immune cell function	● Investigate the interplay between ROS and actin in neutrophil extracellular trap (NET) formation and its implications for autoimmune diseases. ● Study how actin-ROS interactions influence antigen presentation in dendritic cells or macrophages.
Actin-related mechanisms in aging	● Elucidate how cytoskeleton oxidation and actin remodeling contributes to age-related cellular senescence and impaired tissue repair. ● Investigate potential interventions targeting ROS-actin pathways to enhance regenerative capacity in aging tissues. ● Examine how actin oxidation impacts profilin function and its relevance to neurodegenerative disorders such as ALS. ● Explore the role of SCEV in neurodegeneration and whether they can mitigate disease progression through profilin or other cargo delivery.
Redox mechanisms from cancer initiation to metastasis development	● We were the first to report the mitotic activity of transformed cells ROS production. Studying DNA Damage Response (DDR) in the presence of ROS may may reveal novel mechanisms for tumor progression ● Investigate how redox interventions could impair metastatic potential while sparing normal tissue repair. ● Assess how MICAL1 and MICAL2 expression impacts cancer progression and metastasis.
Synergistic roles of ROS and RNS	● Explore cooperative and antagonistic effects of ROS and RNS on actin cytoskeleton during inflammation and repair. ● Identify actin-regulating enzymes responsive to both ROS and RNS.
ROS and nuclear actin	● Investigate the impact of ROS on Nuclear Actin Structures; for example, how ROS modify nuclear actin to influence transcription factor interactions. ● Study how ROS affect actin-containing chromatin remodeling complexes, such as SWI/SNF or INO80. ● Explore how ROS-induced actin dynamics affect histone modifications, such as acetylation, methylation, and phosphorylation.

## CRediT authorship contribution statement

**Pascal J. Goldschmidt-Clermont:** Writing – review & editing, Writing – original draft, Validation, Supervision, Resources, Conceptualization. **Brock A. Sevilla:** Writing – review & editing, Validation, Software, Conceptualization.

## Declaration of competing interest

The authors do not have a conflict of interest.

## Data Availability

This is a review article and data has been published

## References

[1] Pollard T.D., Cooper J.A. (2009). Actin, a central player in cell shape and movement. Science.

[2] Dominguez R., Holmes K.C. (2011). Actin structure and function. Annu. Rev. Biophys..

[3] Pollard T.D., Borisy G.G. (2003). Cellular motility driven by assembly and disassembly of actin filaments. Cell.

[4] Herrmann J.M., Dick T.P. (2012). Redox biology on the rise. bchm.

[5] Rouyère C., Serrano T., Frémont S., Echard A. (2022). Oxidation and reduction of actin: origin, impact in vitro and functional consequences in vivo. Eur. J. Cell Biol..

[6] Adams L., Franco M.C., Estevez A.G. (2015). Reactive nitrogen species in cellular signaling. Exp. Biol. Med..

[7] Lushchak V.I. (2014). Free radicals, reactive oxygen species, oxidative stress and its classification. Chem. Biol. Interact..

[8] Valko M., Leibfritz D., Moncol J., Cronin M.T.D., Mazur M., Telser J. (2007). Free radicals and antioxidants in normal physiological functions and human disease. Int. J. Biochem. Cell Biol..

[9] Iyengar M.R., Weber H.H. (1964). The relative affinities of nucleotides to G-actin and their effects. Biochim. Biophys. Acta BBA - Gen Subj.

[10] Varland S., Vandekerckhove J., Drazic A. (2019). Actin post-translational modifications: the cinderella of cytoskeletal control. Trends Biochem. Sci..

[11] Wu W., Hale A.J., Simone L., Den Hertog J. (2017). Differential oxidation of protein-tyrosine phosphatases during zebrafish caudal fin regeneration. Sci. Rep..

[12] Love N.R., Chen Y., Ishibashi S. (2013). Amputation-induced reactive oxygen species are required for successful Xenopus tadpole tail regeneration. Nat. Cell Biol..

[13] Moldovan L., Mythreye K., Goldschmidt-Clermont P.J., Satterwhite L. (2006). Reactive oxygen species in vascular endothelial cell motility. Roles of NAD(P)H oxidase and Rac1. Cardiovasc. Res..

[14] Sen C.K., Khanna S., Babior B.M., Hunt T.K., Ellison E.C., Roy S. (2002). Oxidant-induced vascular endothelial growth factor expression in human keratinocytes and cutaneous wound healing. J. Biol. Chem..

[15] Dos Remedios C.G., Chhabra D., Kekic M. (2003). Actin binding proteins: regulation of cytoskeletal microfilaments. Physiol. Rev..

[16] Chesarone M.A., Goode B.L. (2009). Actin nucleation and elongation factors: mechanisms and interplay. Curr. Opin. Cell Biol..

[17] Patel K.D., Keskin-Erdogan Z., Sawadkar P. (2024). Oxidative stress modulating nanomaterials and their biochemical roles in nanomedicine. Nanoscale Horiz.

[18] Sundaresan M., Yu Z.X., Ferrans V.J., Irani K., Finkel T. (1995). Requirement for generation of H_2_ O_2_ for platelet-derived growth factor signal transduction. Science.

[19] Kwon J., Lee S.R., Yang K.S. (2004). Reversible oxidation and inactivation of the tumor suppressor PTEN in cells stimulated with peptide growth factors. Proc. Natl. Acad. Sci..

[20] Halvey P.J., Watson W.H., Hansen J.M., Go Y.M., Samali A., Jones D.P. (2005). Compartmental oxidation of thiol–disulphide redox couples during epidermal growth factor signalling. Biochem. J..

[21] Sirokmány G., Pató A., Zana M. (2016). Epidermal growth factor-induced hydrogen peroxide production is mediated by dual oxidase 1. Free Radic. Biol. Med..

[22] Pollard T.D., Blanchoin L., Mullins R.D. (2000). Molecular mechanisms controlling actin filament dynamics in nonmuscle cells. Annu. Rev. Biophys. Biomol. Struct..

[23] Goldschmidt-Clermont P.J., Furman M.I., Wachsstock D., Safer D., Nachmias V.T., Pollard T.D. (1992). The control of actin nucleotide exchange by thymosin beta 4 and profilin. A potential regulatory mechanism for actin polymerization in cells. Mol. Biol. Cell.

[24] Lassing I., Schmitzberger F., Björnstedt M. (2007). Molecular and structural basis for redox regulation of β-actin. J. Mol. Biol..

[25] Pinto‐Costa R., Sousa M.M. (2020). Profilin as a dual regulator of actin and microtubule dynamics. Cytoskeleton.

[26] Jockusch B.M., Murk K., Rothkegel M. (2007). The profile of profilins. Rev. Physiol. Biochem. Pharmacol..

[27] Machesky L.M., Insall R.H. (1998). Scar1 and the related Wiskott–Aldrich syndrome protein, WASP, regulate the actin cytoskeleton through the Arp2/3 complex. Curr. Biol..

[28] Goldschmidt-Clermont P.J., Kim J.W., Machesky L.M., Rhee S.G., Pollard T.D. (1991). Regulation of phospholipase C-γ1 by profilin and tyrosine phosphorylation. Science.

[29] Finkel T., Theriot J.A., Dise K.R., Tomaselli G.F., Goldschmidt-Clermont P.J. (1994). Dynamic actin structures stabilized by profilin. Proc. Natl. Acad. Sci..

[30] Small J.V., Stradal T., Vignal E., Rottner K. (2002). The lamellipodium: where motility begins. Trends Cell Biol..

[31] Lou S.S., Kennard A.S., Koslover E.F., Gutierrez E., Groisman A., Theriot J.A. (2021). Elastic wrinkling of keratocyte lamellipodia driven by myosin-induced contractile stress. Biophys. J..

[32] Stroka K.M., Jiang H., Chen S.H. (2014). Water permeation drives tumor cell migration in confined microenvironments. Cell.

[33] Vasiliev J.M. (1991). Polarization of pseudopodial activities: cytoskeletal mechanisms. J. Cell Sci..

[34] Pollard T.D., Machesky L., Goldschmidt-Clermont P. (1991). Current Topics in Membranes.

[35] Goldschmidt-Clermont P.J., Hubinont C., Goldschmidt A.J.P., DiFede D.L., White I.A. (2021). Pregnancy, a unique case of heterochronic parabiosis and peripartum cardiomyopathy. Front Biosci Landmark.

[36] Crawford L.E., Milliken E.E., Irani K. (1996). Superoxide-mediated actin response in post-hypoxic endothelial cells. J. Biol. Chem..

[37] Moldovan L., Irani K., Moldovan N.I., Finkel T., Goldschmidt-Clermont P.J. (1999). The actin cytoskeleton reorganization induced by Rac1 requires the production of superoxide. Antioxidants Redox Signal..

[38] Goldschmidt-Clermont P.J., Mendelsohn M.E., Gibbs J.B. (1992). Rac and Rho in control. Curr. Biol..

[39] Ridley A.J., Paterson H.F., Johnston C.L., Diekmann D., Hall A. (1992). The small GTP-binding protein rac regulates growth factor-induced membrane ruffling. Cell.

[40] Rhee S.G. (2006). H_2_ O_2_ , a necessary evil for cell signaling. Science.

[41] Lindberg U., Karlsson R., Lassing I., Schutt C.E., Höglund A.S. (2008). The microfilament system and malignancy. Semin. Cancer Biol..

[42] Moldovan L., Moldovan N.I., Sohn R.H., Parikh S.A., Goldschmidt-Clermont P.J. (2000). Redox changes of cultured endothelial cells and actin dynamics. Circ. Res..

[43] Irani K., Xia Y., Zweier J.L. (1997). Mitogenic signaling mediated by oxidants in ras-transformed fibroblasts. Science.

[44] Suh Y.A., Arnold R.S., Lassegue B. (1999). Cell transformation by the superoxide-generating oxidase Mox1. Nature.

[45] Mitsushita J., Lambeth J.D., Kamata T. (2004). The superoxide-generating oxidase Nox1 is functionally required for ras oncogene transformation. Cancer Res..

[46] Ma Q., Cavallin L.E., Yan B. (2009). Antitumorigenesis of antioxidants in a transgenic Rac1 model of Kaposi's sarcoma. Proc. Natl. Acad. Sci..

[47] Herrero-Cervera A., Soehnlein O., Kenne E. (2022). Neutrophils in chronic inflammatory diseases. Cell. Mol. Immunol..

[48] Surmi B., Hasty A. (2008). Macrophage infiltration into adipose tissue: initiation, propagation and remodeling. Future Lipidol..

[49] Fletcher J.W., Djulbegovic B., Soares H.P. (2008). Recommendations on the use of^18^ F-FDG PET in oncology. J. Nucl. Med..

[50] Narrated (2013). Membrane ruffling and intracellular vacuoles. https://www.youtube.com/watch?v=1LumwOqaJgY.

[51] Bar-Sagi D., Feramisco J.R. (1986). Induction of membrane ruffling and fluid-phase pinocytosis in quiescent fibroblasts by *ras* proteins. Science.

[52] Commisso C., Davidson S.M., Soydaner-Azeloglu R.G. (2013). Macropinocytosis of protein is an amino acid supply route in Ras-transformed cells. Nature.

[53] Garcia-Bermudez J., Badgley M.A., Prasad S. (2022). Adaptive stimulation of macropinocytosis overcomes aspartate limitation in cancer cells under hypoxia. Nat. Metab..

[54] Khan A., Oliveira J., Lee Y.S. (2025). Human Schwann cell-derived extracellular vesicle isolation, bioactivity assessment, and omics characterization. Int. J. Nanomed..

[55] Fan M., Wu H., Sferruzzi-Perri A.N., Wang Y.L., Shao X. (2024). Endocytosis at the maternal-fetal interface: balancing nutrient transport and pathogen defense. Front. Immunol..

[56] Canton J. (2018). Macropinocytosis: new insights into its underappreciated role in innate immune cell surveillance. Front. Immunol..

[57] Steinman R.M., Brodie S.E., Cohn Z.A. (1976). Membrane flow during pinocytosis. A stereologic analysis. J. Cell Biol..

[58] Giannoni E., Buricchi F., Raugei G., Ramponi G., Chiarugi P. (2005). Intracellular reactive oxygen species activate Src tyrosine kinase during cell adhesion and anchorage-dependent cell growth. Mol. Cell Biol..

[59] Tehrani S., Tomasevic N., Weed S., Sakowicz R., Cooper J.A. (2007). Src phosphorylation of cortactin enhances actin assembly. Proc. Natl. Acad. Sci..

[60] Liu T., Cao L., Mladenov M., Jegou A., Way M., Moores C.A. (2024). Cortactin stabilizes actin branches by bridging activated Arp2/3 to its nucleated actin filament. Nat. Struct. Mol. Biol..

[61] Wang J., Boja E.S., Tan W. (2001). Reversible glutathionylation regulates actin polymerization in A431 cells. J. Biol. Chem..

[62] Choudhary B.S., Chaudhary N., Shah M. (2023). Lipocalin 2 inhibits actin glutathionylation to promote invasion and migration. FEBS Lett..

[63] Sakai J., Li J., Subramanian K.K. (2012). Reactive oxygen species-induced actin glutathionylation controls actin dynamics in neutrophils. Immunity.

[64] Chen F.C., Ogut O. (2006). Decline of contractility during ischemia-reperfusion injury: actin glutathionylation and its effect on allosteric interaction with tropomyosin. Am. J. Physiol. Cell Physiol..

[65] Burns M., Rizvi S.H.M., Tsukahara Y. (2020). Role of glutaredoxin-1 and glutathionylation in cardiovascular diseases. Int. J. Mol. Sci..

[66] Kruyer A., Ball L.E., Townsend D.M., Kalivas P.W., Uys J.D., Cardoso Cruz F. (2019). Post-translational S-glutathionylation of cofilin increases actin cycling during cocaine seeking. PLoS One.

[67] Rodriguez A., Kashina A. (2018). Posttranscriptional and posttranslational regulation of actin. Anat. Rec..

[68] Liou G.Y., Storz P. (2010). Reactive oxygen species in cancer. Free Radic. Res..

[69] Perillo B., Di Donato M., Pezone A. (2020). ROS in cancer therapy: the bright side of the moon. Exp. Mol. Med..

[70] Zhou J., Zhang L., Zeng L. (2021). Helicobacter pylori FabX contains a [4Fe-4S] cluster essential for unsaturated fatty acid synthesis. Nat. Commun..

[71] Dröge W. (2002). Free radicals in the physiological control of cell function. Physiol. Rev..

[72] Fearon E.R., Vogelstein B. (1990). A genetic model for colorectal tumorigenesis. Cell.

[73] Rhee S.G., Bae Y.S., Lee S.R., Kwon J. (2000). Hydrogen peroxide: a key messenger that modulates protein phosphorylation through cysteine oxidation. Sci. STKE.

[74] Mellström K., Heldin C.H., Westermark B. (1988). Induction of circular membrane ruffling on human fibroblasts by platelet-derived growth factor. Exp. Cell Res..

[75] Puccini J., Wei J., Tong L., Bar-Sagi D. (2023). Cytoskeletal association of ATP citrate lyase controls the mechanodynamics of macropinocytosis. Proc. Natl. Acad. Sci..

[76] Tannetta D., Dragovic R., Alyahyaei Z., Southcombe J. (2014). Extracellular vesicles and reproduction–promotion of successful pregnancy. Cell. Mol. Immunol..

[77] Terman J.R., Kashina A. (2013). Post-translational modification and regulation of actin. Curr. Opin. Cell Biol..

[78] Thom S.R., Bhopale V.M., Mancini D.J., Milovanova T.N. (2008). Actin S-nitrosylation inhibits neutrophil β2 integrin function. J. Biol. Chem..

[79] Thom S.R., Bhopale V.M., Yang M., Bogush M., Huang S., Milovanova T.N. (2011). Neutrophil β2 integrin inhibition by enhanced interactions of vasodilator-stimulated phosphoprotein with S-nitrosylated actin. J. Biol. Chem..

[80] Vicente-Manzanares M., Choi C.K., Horwitz A.R. (2009). Integrins in cell migration – the actin connection. J. Cell Sci..

[81] Thom S.R., Bhopale V.M., Yang M. (2014). Neutrophils generate microparticles during exposure to inert gases due to cytoskeletal oxidative stress. J. Biol. Chem..

[82] Yoon J., Terman J.R. (2018). MICAL redox enzymes and actin remodeling: new links to classical tumorigenic and cancer pathways. Mol. Cell. Oncol..

[83] Mariotti S., Barravecchia I., Vindigni C. (2016). *MICAL2* is a novel human cancer gene controlling mesenchymal to epithelial transition involved in cancer growth and invasion. Oncotarget.

[84] McGarry D.J., Armstrong G., Castino G. (2021). MICAL1 regulates actin cytoskeleton organization, directional cell migration and the growth of human breast cancer cells as orthotopic xenograft tumours. Cancer Lett..

[85] Johansson M., Lundberg M. (2007). Glutathionylation of beta-actin via a cysteinyl sulfenic acid intermediary. BMC Biochem..

[86] Fiaschi T., Cozzi G., Raugei G., Formigli L., Ramponi G., Chiarugi P. (2006). Redox regulation of β-actin during integrin-mediated cell adhesion. J. Biol. Chem..

[87] Hattori H., Subramanian K.K., Sakai J. (2010). Small-molecule screen identifies reactive oxygen species as key regulators of neutrophil chemotaxis. Proc. Natl. Acad. Sci..

[88] Kalinina E., Novichkova M. (2021). Glutathione in protein redox modulation through S-glutathionylation and S-nitrosylation. Molecules.

[89] Uemura T., Tsaprailis G., Gerner E.W. (2019). GSTΠ stimulates caveolin-1-regulated polyamine uptake via actin remodeling. Oncotarget.

[90] Galeazzi R., Laudadio E., Falconi E. (2018). Protein–protein interactions of human glyoxalase II: findings of a reliable docking protocol. Org. Biomol. Chem..

[91] Pizarro G.O., Ogut O. (2009). Impact of actin glutathionylation on the actomyosin-S1 ATPase. Biochemistry.

[92] Kommaddi R.P., Karunakaran S., Tomar D.S., Ray A., Ravindranath V. (2018). P3‐150: glutaredoxin1 overexpression ameliorates early F‐actin loss and cognitive deficits seen in ALZHEIMER’S disease mouse model. Alzheimers Dement.

[93] Negre-Salvayre A., Guerby P., Gayral S., Laffargue M., Salvayre R. (2020). Role of reactive oxygen species in atherosclerosis: lessons from murine genetic models. Free Radic. Biol. Med..

[94] Li H., Horke S., Förstermann U. (2014). Vascular oxidative stress, nitric oxide and atherosclerosis. Atherosclerosis.

[95] Goldschmidt-Clermont P.J., Creager M.A., Lorsordo D.W., Lam G.K.W., Wassef M., Dzau V.J. (2005). Atherosclerosis 2005: recent discoveries and novel hypotheses. Circulation.

[96] Head T., Daunert S., Goldschmidt-Clermont P.J. (2017). The aging risk and atherosclerosis: a fresh look at arterial homeostasis. Front. Genet..

[97] Najjar S.S., Scuteri A., Lakatta E.G. (2005). Arterial aging: is it an immutable cardiovascular risk factor?. Hypertension.

[98] Herrington W., Lacey B., Sherliker P., Armitage J., Lewington S. (2016). Epidemiology of atherosclerosis and the potential to reduce the global burden of atherothrombotic disease. Circ. Res..

[99] Rauscher F.M., Goldschmidt-Clermont P.J., Davis B.H. (2003). Aging, progenitor cell exhaustion, and atherosclerosis. Circulation.

[100] Karra R., Vemullapalli S., Dong C. (2005). Molecular evidence for arterial repair in atherosclerosis. Proc. Natl. Acad. Sci..

[101] Gu Y., Filippi M.D., Cancelas J.A. (2003). Hematopoietic cell regulation by Rac1 and Rac2 guanosine triphosphatases. Science.

[102] Ballard V.L.T., Edelberg J.M. (2007). Stem cells and the regeneration of the aging cardiovascular system. Circ. Res..

[103] Irani K. (2000). Oxidant signaling in vascular cell growth, death, and survival: a review of the roles of reactive oxygen species in smooth muscle and endothelial cell mitogenic and apoptotic signaling. Circ. Res..

[104] Burtenshaw D., Kitching M., Redmond E.M., Megson I.L., Cahill P.A. (2019). Reactive oxygen species (ROS), intimal thickening, and subclinical atherosclerotic disease. Front. Cardiovasc. Med..

[105] Yung L., Leung F., Yao X., Chen Z.Y., Huang Y. (2006). Reactive oxygen species in vascular wall. Cardiovasc. Hematol. Disord.: Drug Targets.

[106] Stossel T.P., Hartwig J.H., Janmey P.A., Kwiatkowski D.J. (1999). Cell crawling two decades after Abercrombie. Biochem. Soc. Symp..

[107] Grintsevich E.E., Ge P., Sawaya M.R. (2017). Catastrophic disassembly of actin filaments via Mical-mediated oxidation. Nat. Commun..

[108] Hung R.J., Spaeth C.S., Yesilyurt H.G., Terman J.R. (2013). SelR reverses Mical-mediated oxidation of actin to regulate F-actin dynamics. Nat. Cell Biol..

[109] Selvakumar B., Hess D.T., Goldschmidt-Clermont P.J., Stamler J.S. (2008). Co‐regulation of constitutive nitric oxide synthases and NADPH oxidase by the small GTPase Rac. FEBS Lett..

[110] Liu X., Nie L., Zhang Y. (2023). Actin cytoskeleton vulnerability to disulfide stress mediates disulfidptosis. Nat. Cell Biol..

[111] Wioland H., Frémont S., Guichard B., Echard A., Jégou A., Romet‐Lemonne G. (2021). Actin filament oxidation by MICAL1 suppresses protections from cofilin‐induced disassembly. EMBO Rep..

[112] Bernstein B.W., Shaw A.E., Minamide L.S., Pak C.W., Bamburg J.R. (2012). Incorporation of cofilin into rods depends on disulfide intermolecular bonds: implications for actin regulation and neurodegenerative disease. J. Neurosci..

[113] Whiteman I.T., Gervasio O.L., Cullen K.M. (2009). Activated actin-depolymerizing factor/cofilin sequesters phosphorylated microtubule-associated protein during the assembly of alzheimer-like neuritic cytoskeletal striations. J. Neurosci..

[114] McGarry D.J., Castino G., Lilla S., Zanivan S., Olson M.F. (2021).

[115] Krishnan K., Moens P.D.J. (2009). Structure and functions of profilins. Biophys. Rev..

[116] Goldschmidt-Clermont P.J., Khan A., Jimsheleishvili G. (2025). Treating amyotrophic lateral sclerosis with allogeneic Schwann cell–derived exosomal vesicles: a case report. Neural Regen Res..

[117] Montani L., Buerki-Thurnherr T., De Faria J.P. (2014). Profilin 1 is required for peripheral nervous system myelination. Development.

